# Supplementary material for: Anthelmintic resistance against benzimidazoles and macrocyclic lactones in strongyle populations on cattle farms in northern Germany
Source: Sci Rep. 2025 May 23;15:17973. doi: 10.1038/s41598-025-02838-7 (PMC12102382; doi:10.1038/s41598-025-02838-7)
Supplement: Supplementary file 1 — Supplementary Figure S1. [file 41598_2025_2838_MOESM1_ESM.pdf]

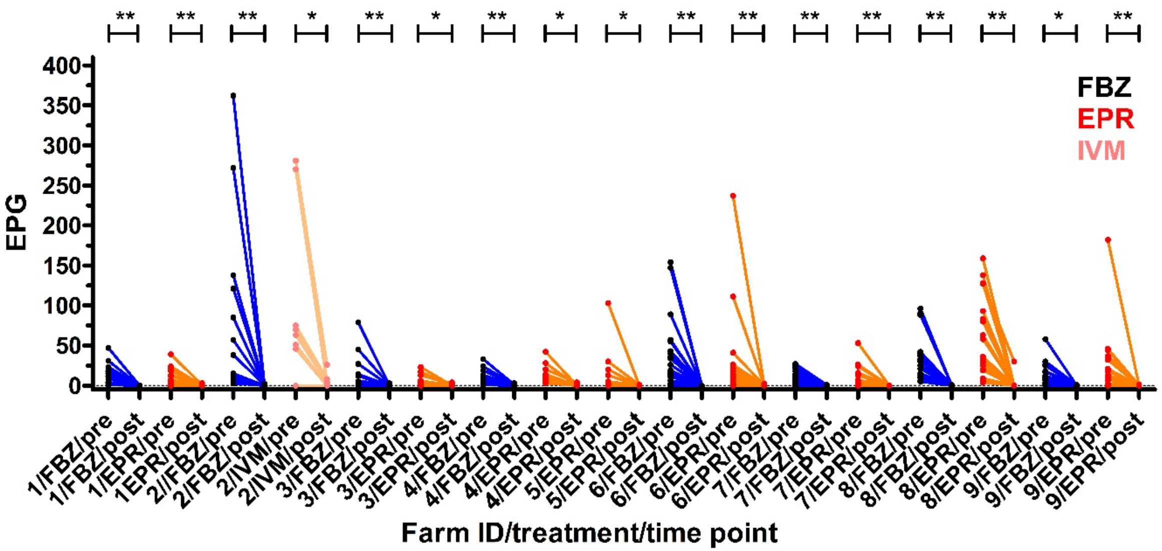

Fig S1. Fecal egg counts in eggs per gram feces (EPG) before and after treatment. Pre-treatment samples were collected from the animals on the day of treatment with either fenbendazole (FBZ), eprinomectin (EPR) or ivermectin (IVM). Egg counts of animals positive for strongyle eggs before treatment and the same animals 14 days post treatment were compared with the Wilcoxon matched-pairs signed rank test. **, $p < 0.01$; *, $p < 0.05$. All p values in Fig. S1 and Fig S2 were corrected together for multiple testing using the Holm-Bonferroni method.
