# Supplementary material for: Anthelmintic resistance against benzimidazoles and macrocyclic lactones in strongyle populations on cattle farms in northern Germany
Source: Sci Rep. 2025 May 23;15:17973. doi: 10.1038/s41598-025-02838-7 (PMC12102382; doi:10.1038/s41598-025-02838-7)
Supplement: Supplementary file 2 — Supplementary Figure S2. [file 41598_2025_2838_MOESM2_ESM.pdf]

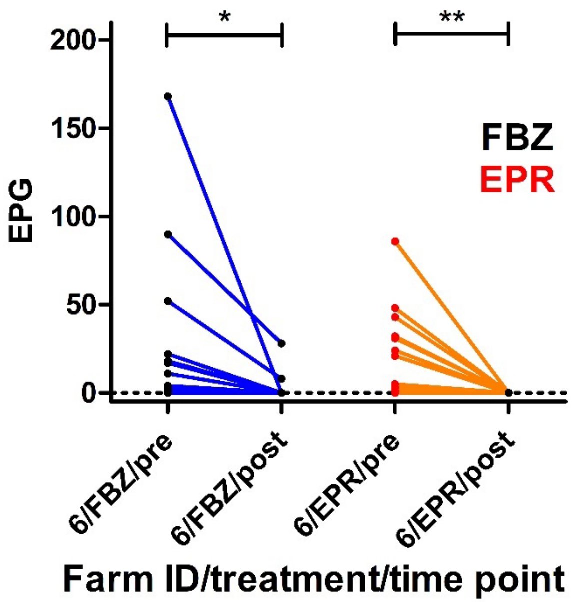

Fig. S2. Fecal egg counts in *Nematodirus* spp. eggs per gram feces (EPG) before and after treatment. Pre-treatment samples were collected from the animals on the day of treatment with either fenbendazole (FBZ) or eprinomectin (EPR). Egg counts of animals positive for *Nematodirus spp*. eggs before treatment and the same animals 14 days post treatment were compared with the Wilcoxon matched-pairs signed rank test. **, $p < 0.01$; *, $p < 0.05$. All p values in Fig. S1 and Fig S2 were corrected together for multiple testing using the Holm-Bonferroni method.
