# Supplementary material for: Anthelmintic resistance against benzimidazoles and macrocyclic lactones in strongyle populations on cattle farms in northern Germany
Source: Sci Rep. 2025 May 23;15:17973. doi: 10.1038/s41598-025-02838-7 (PMC12102382; doi:10.1038/s41598-025-02838-7)
Supplement: Supplementary file 3 — Supplementary Figure S3. [file 41598_2025_2838_MOESM3_ESM.pdf]

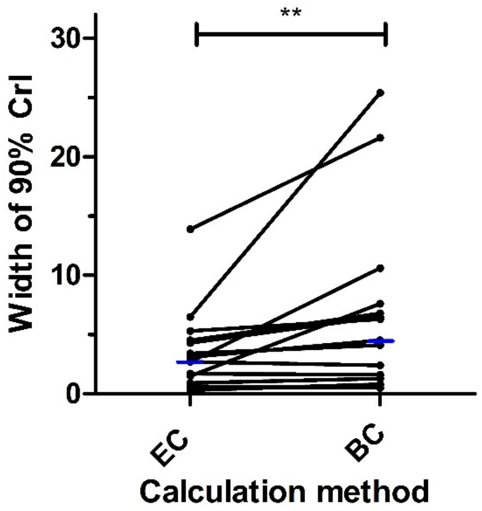

Fig. S3. Comparison of the width of the 90% credible intervals (CrIs) between eggCounts (EC) and bayescounts (BC) statistical approaches. Results for the same dataset are connected by lines. Blue lines indicate the medians. Data were compared using a Wilcoxon matched-pairs rank signed test. **, $p < 0.01$.
