# Supplementary material for: Anthelmintic resistance against benzimidazoles and macrocyclic lactones in strongyle populations on cattle farms in northern Germany
Source: Sci Rep. 2025 May 23;15:17973. doi: 10.1038/s41598-025-02838-7 (PMC12102382; doi:10.1038/s41598-025-02838-7)
Supplement: Supplementary file 4 — Supplementary Table S1. [file 41598_2025_2838_MOESM4_ESM.pdf]

Primer names	Primer sequences (5' -> 3')
Forward primers[a]	
NC1_with_Illumina_Adapter_(0N)	TCGTCGGCAGCGTCAGATGTGTATAAGAGACAGACGTCTGGTTCAGGGTTGTT
NC1_with_Illumina_Adapter_(1N)	TCGTCGGCAGCGTCAGATGTGTATAAGAGACAGNACGTCTGGTTCAGGGTTGTT
NC1_with_Illumina_Adapter_(2N)	TCGTCGGCAGCGTCAGATGTGTATAAGAGACAGNNACGTCTGGTTCAGGGTTGTT
NC1_with_Illumina_Adapter_(3N)	TCGTCGGCAGCGTCAGATGTGTATAAGAGACAGNNNACGTCTGGTTCAGGGTTGTT
Reverse primers[a]	
NC2_with_Illumina_Adapter_(0N)	GTCTCGTGGGCTCGGAGATGTGTATAAGAGACAGTTAGTTTCTTTTCCTCCGCT
NC2_with_Illumina_Adapter_(1N)	GTCTCGTGGGCTCGGAGATGTGTATAAGAGACAGNTTAGTTTCTTTTCCTCCGCT
NC2_with_Illumina_Adapter_(2N)	GTCTCGTGGGCTCGGAGATGTGTATAAGAGACAGNNTTAGTTTCTTTTCCTCCGCT
NC2_with_Illumina_Adapter_(3N)	GTCTCGTGGGCTCGGAGATGTGTATAAGAGACAGNNNTTAGTTTCTTTTCCTCCGCT

Table S1 Primers for amplification of internal transcribed spacer for Illumina sequencing

[a]In order to avoid problems with calculation of correction factors, which is not possible if the same base is detected for all positions on the Illumina Flow Cell during primer sequencing, zero to three random bases ($N_{0\text{-}3}$) were inserted between the NC1/NC2 sequences and the Illumina adapters, as detailed by Avramenko, et al.[23]
