# Supplementary material for: Anthelmintic resistance against benzimidazoles and macrocyclic lactones in strongyle populations on cattle farms in northern Germany
Source: Sci Rep. 2025 May 23;15:17973. doi: 10.1038/s41598-025-02838-7 (PMC12102382; doi:10.1038/s41598-025-02838-7)
Supplement: Supplementary file 5 — Supplementary Table S2. [file 41598_2025_2838_MOESM5_ESM.pdf]

Species	N positive	Prevalence (%)	95% CI (%)
Cooperia oncophora	8	88.9	56.5 – 98.0
Cooperia punctata	5	55.6	26.7 – 81.1
Cooperia spp.	5	55.6	26.7 – 81.1
Ostertagia ostertagi	9	100	70.1 - 100
Ostertagia leptospicularis	2	22.2	6.3 – 54.7
Oesophagostomum radiatum	4	44.4	18.9 – 73.3
Oesophagostomum venulosum	4	44.4	18.9 – 73.3
Trichostrongylus axei	4	44.4	18.9 – 73.3
Trichostrongylus colubriformis	1	11.1	2.0 – 43.5
Trichostrongylus spp.	2	22.2	6.3 – 54.7
Chabertia ovina	1	11.1	2.0 – 43.5
Bunostomum phlebotomum	3	33.3	12.1 – 64.6
Haemonchus contortus	1	11.1	2.0 – 43.5
Strongylidae	1	11.1	2.0 – 43.5

Table S2 Farm prevalence with 95% confidence intervals (CI) of strongyle species identified by deep amplicon sequences in nine German cattle farms before treatment.
